# The effect of heat treatment on the morphology and mobility of Au nanoparticles

**DOI:** 10.3762/bjnano.11.6

**Published:** 2020-01-06

**Authors:** Sven Oras, Sergei Vlassov, Simon Vigonski, Boris Polyakov, Mikk Antsov, Vahur Zadin, Rünno Lõhmus, Karine Mougin

**Affiliations:** 1 Institute of Physics, University of Tartu, W. Ostwaldi tn 1, 50412, Tartu, Estonia; 2 Université de Strasbourg, Université de Haute Alsace, Institut de Science des Matériaux, IS2M-CNRS-UMR 7361, 15 Rue Jean Starcky, 68057 Mulhouse, France; 3 Tallinn University of Technology, Tartu College, Puiestee 78, Tartu, 51008, Estonia; 4 Institute of Technology, University of Tartu, Nooruse 1, 50411, Tartu, Estonia; 5 Institute of Solid State Physics, University of Latvia, Kengaraga street 8, LV-1063 Riga, Latvia

**Keywords:** annealing, atomic force microscopy (AFM), Au nanoparticles, manipulation, melting, nanotribology

## Abstract

In the present paper, we investigate the effect of heat treatment on the geometry and mobility of Au nanoparticles (NPs) on a Si substrate. Chemically synthesized Au NPs of diameter ranging from 5 to 27 nm were annealed at 200, 400, 600 and 800 °C for 1 h. A change in the geometry from faceted to more rounded shapes were observed with increasing annealing temperature. Kinetic Monte Carlo simulations indicate that the NPs become rounded due to the minimization of the surface area and the transition to lower energy surface types {111} and {100}. The NPs were manipulated on a silica substrate with an atomic force microscope (AFM) in tapping mode. Initially, the NPs were immovable by AFM energy dissipation. However, annealed NPs became movable, and less energy was required to displace the NPs annealed at higher temperature. However, after annealing at 800 °C, the particles became immovable again. This effect was attributed to the diffusion of Au into the Si substrate and to the growth of the SiO_2_ layer.

## Introduction

Gold is one of the most prominent materials used in studies related to nanostructures. The small size and the enhanced properties of Au nanoparticles (NPs) compared to bulk gold make them important for the development of novel applications, for example, in the field of drug delivery [1], sensor technology [2], printing [3] and catalysis [4]. Due to their inert state, geometrical diversity and convenient synthesis, Au NPs are an attractive model system for nanotribological manipulation experiments [5–12] and simulations [13]. Additional flexibility is provided by the ability to tune the properties of the NPs by varying the size [14]_,_ shape [14] and chemical composition of the outer layer covering the NP [5]. These modifications become especially important for tribological applications since they can affect the interfacial behavior during contact.

The shape of a NP is known to have a significant impact on the contact area between the NP and a substrate [14]. The morphology, and thus the contact area of the NP, can be tuned by changing the synthesis parameters [15]. For readily synthesized NPs, the morphology can be changed by adding energy to the NPs by laser treatment [16] or by heating the NPs in an oven. Partial melting can be achieved at a significantly lower temperature than the melting temperature of bulk Au [17].

In this study, chemically synthesized faceted Au NPs were annealed at different temperatures between 200 and 800 °C, which resulted in rounding of the NPs. The particles were then displaced with an atomic force microscope (AFM) in order to study the effect of annealing on their tribological behavior.

## Experimental

### Nanoparticle synthesis

A colloidal suspension of Au NPs was produced by reducing an aqueous solution of HAuCl_4_·H_2_O. The procedure consisted of adding 3 mL of 1% aqueous HAuCl_4_ (Sigma-Aldrich) to 150 mL of pure H_2_O at 90 °C under vigorous stirring. After 1 min, 2 mL of a 1% aqueous solution of sodium citrate (C_6_H_5_Na_3_O_7_, Sigma-Aldrich) were added to stabilize the suspension. By reducing HAuCl_4_, sodium citrate transfers the negative charge of the citrate ions to the Au NP surface [18–19]. The solution was stirred for 5 min and then stored at 4 °C until needed [10].

### Annealing

The silicon wafers (100, n/phosphorus-doped, 3–6 ohm·cm, Mat-Technology) were cleaned with ethanol and the NPs were deposited by drop-casting. The drops were dried at ambient conditions. Five samples of Au NPs on Si wafers were prepared, and four of them were then separately annealed in an oven for 1 h at 200, 400, 600 and 800 °C while the fifth was left untreated. Separate samples were prepared on heat-resistant silicon nitride support films of 50 nm membrane thickness (Pelco, Ted Pella) to be used for transmission electron microscopy (TEM).

### Characterization

The morphology of the NPs annealed at different temperatures was studied by TEM (ARM200, JEOL). A null ellipsometer (Multiskop, Optrel, Germany) equipped with a 532 nm Nd:YAG laser was used for measuring the thickness of the SiO_2_ layers.

### AFM setup

The manipulation of the NPs was performed with a Bruker Multimode 8 AFM in the PeakForce quantitative nanoscale mechanical characteriztion (PeakForce QNM) mode using a rectangular AFM cantilever (Bruker, RTESPA-300, *k* = 40 N/m) with a resonance frequency of around 300 kHz.

Prior to each manipulation, the samples were heated to 100 °C to remove excess water. An image was first taken in the high-resolution QNM mode to find the Au particles. Then, the operation mode was switched to tapping mode. The oscillation amplitude was kept constant with a feedback loop on, and the power dissipated during tapping was calculated with the following equation [20]:


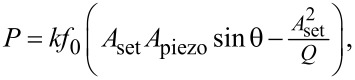


where *k* is the cantilever spring constant, *f*_0_ is the resonance frequency of the cantilever, *A*_set_ is the setpoint amplitude, *A*_piezo_ is the drive amplitude, θ is the phase signal and *Q* is the quality factor of the AFM cantilever. The dissipated power was used as a measure of the mobility of the NPs.

The calibration of the cantilevers was performed by the thermal tuning method. The oscillation amplitudes ranged from 0.05 to 0.6 V with a sensitivity of 25 to 31 nm/V. The sensitivity was measured individually for each cantilever by means of damping the AFM tip against a silicon substrate. The phase values were extracted from the tapping mode phase images for each manipulated NP and processed using the Gwyddion software. The phase shift values were extracted from the profile of the trace on the phase shift image by averaging ten lines using the Gwyddion program.

After each scan, the force applied on the NPs was increased until all the particles in the area were displaced or the force was high enough to start severely damaging the tip. If the tip was too dull for performing manipulations, the tip was replaced with a fresh and sharp AFM tip.

### Simulation setup

Simulations of the NP rounding were carried out using the kinetic Monte Carlo (KMC) code Kimocs [21], which is specifically designed for metal surfaces. The parameters for Au, developed by Vigonski et al. [17], use the tethering method for stabilization [22].

Initially cubic NPs of two sizes with side lengths of 2 and 5 nm were chosen. The simulations were performed at 726.85 °C (1000 K) in order to obtain results within a reasonable computation time. This simplification is expected to have only minor influence, especially in the case of a NP diameter of 5 nm and larger [17]. The parametrization of Au implemented by Vigonski et al. [17] utilizes only nearest-neighbor jumps, which lead to a rather uniform behavior of larger NPs. In the diffusion model used in Kimocs, the computational time increases exponentially with decreasing temperature, making simulations at experimental temperatures unfeasible. Nevertheless, the temperature used in the simulations is significantly below the melting temperature of Au. Furthermore, in the computational model, the dynamics of diffusion are unaffected by temperature, making comparisons to experimental results possible [21]. Visualizations of the results of the simulation were generated with Ovito [23].

## Results and Discussion

**Figure 1:**
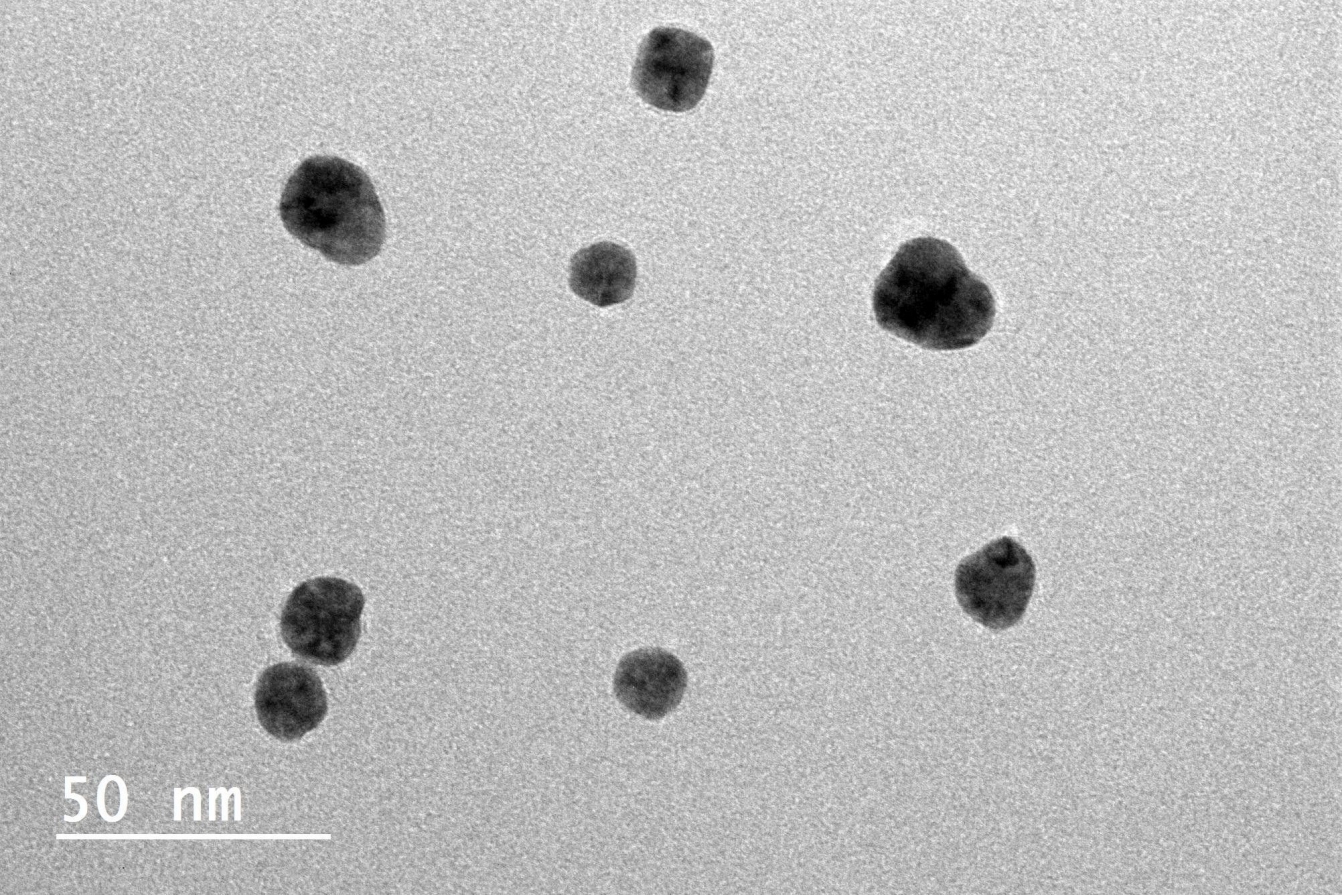
Typical TEM image of Au NPs before thermal treatment.

As observed in the TEM images, most of the untreated Au NPs had irregular faceted shapes with a diameter ranging from 5 to 27 nm with a median diameter of 13 ± 3.96 nm (measured on 90 particles). The term “diameter” in the present study was defined as the average value of the largest and the smallest dimension of the NPs visible on the TEM image. A typical TEM image of the synthesized Au NPs is presented in Figure 1.

### Influence of temperature – change in morphology

**Figure 2:**
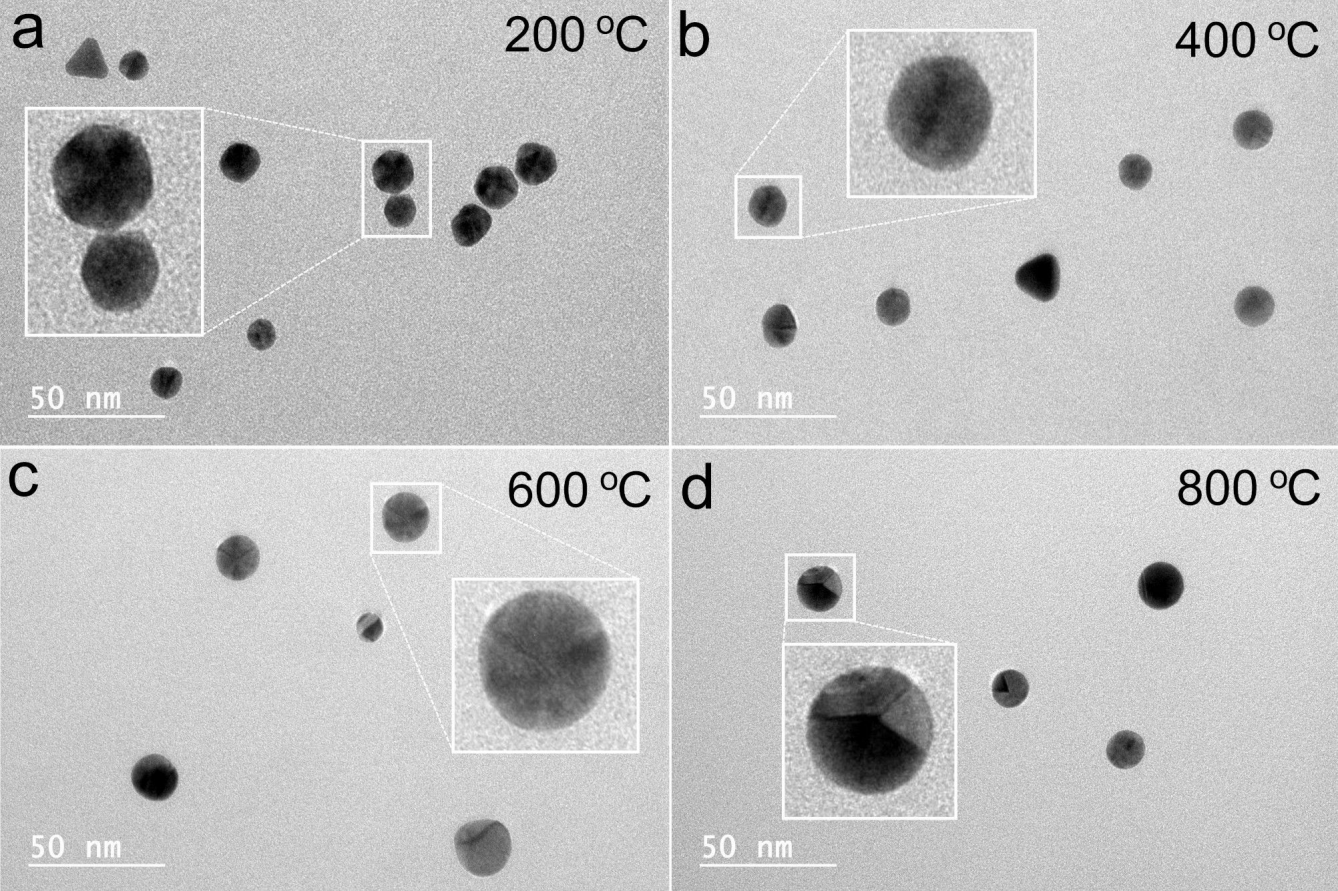
Morphology of the Au NPs and after annealing at (a) 200 (b), 400 (c) 600 (d) and 800 °C. Slight rounding of the NPs can be seen at higher temperatures.

The TEM analysis revealed a considerable change in the geometry of the NPs to a more rounded shape as a result of annealing, although the temperatures used were far below the melting point of bulk Au (1064 °C). Figure 2 presents typical TEM images of the NPs annealed at different temperatures. Some tendency for rounding can be observed even for the sample annealed at 200 °C. The effect becomes clearly visible at 400 °C and increases further for higher temperatures (Table 1). To evaluate the morphological changes more quantitatively, we introduced a criterion based on the length of the facet: if the NP is smaller than 10% of its total visible perimeter, then it is classified as rounded.

**Table 1:** Ratio of truncated to rounded particles depending on the annealing temperature.

Temperature (°C)	Truncated	Rounded	Ratio

200	105	10	10.5
400	64	43	1.5
600	37	42	0.9
800	9	57	0.2

**Figure 3:**
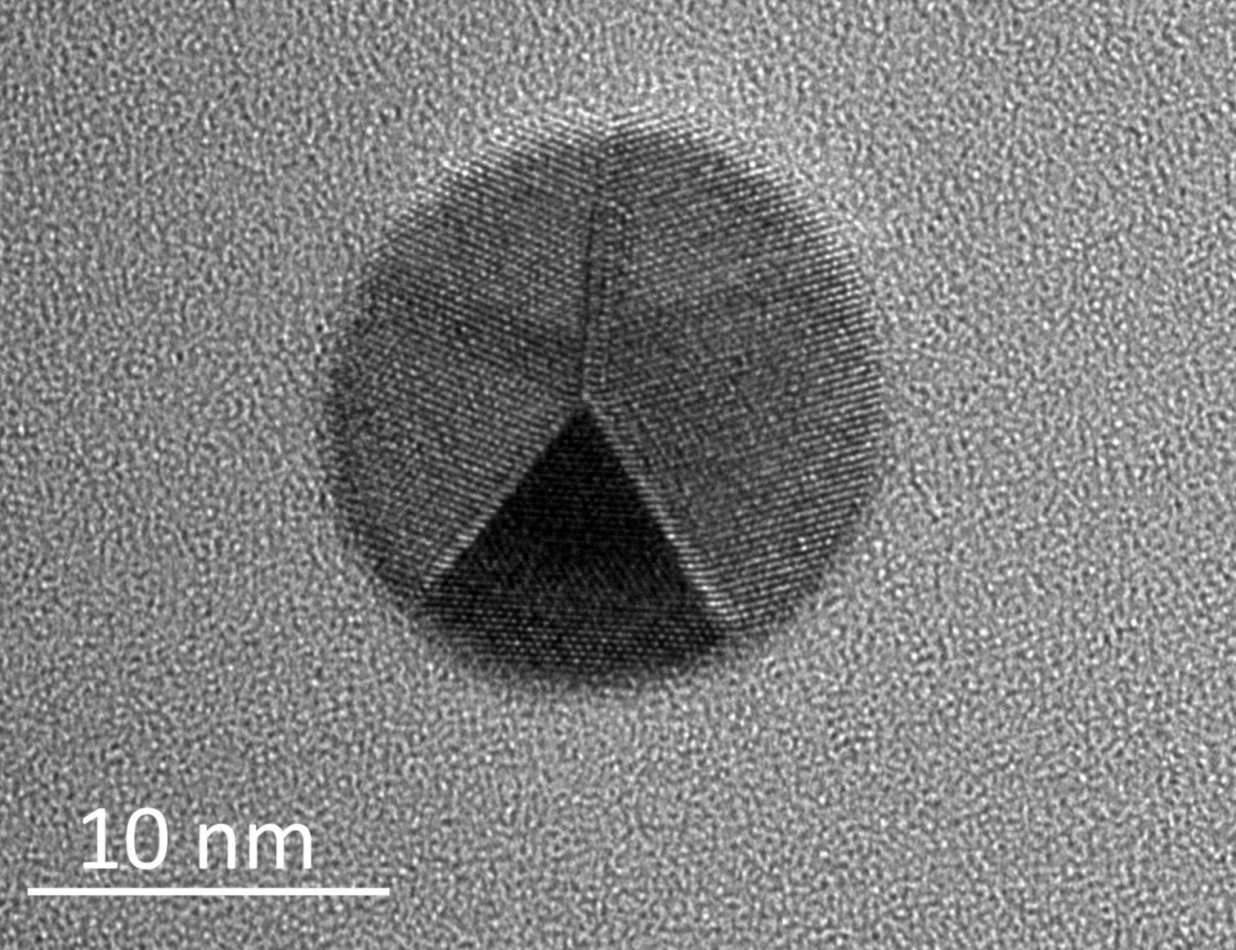
Au NP annealed at 800 °C with well-pronounced five-fold twinned inner structure.

The reduced melting temperature of NPs compared to their bulk counterparts is not a new phenomenon. In 1976, Buffat and Borel [24] demonstrated that the melting temperature of Au NPs is size-dependent. However, the decrease of the melting temperature presented in their work was only up to a few hundred Kelvin for particles of diameter around 5 nm and was even less pronounced for larger particles, approaching the melting point of the bulk above 20 nm. In contrast, we observed rounding at much lower temperatures, even for particles exceeding 20 nm in size. Moreover, rounding of Au NPs at just 500 °C has been reported even for particles exceeding 100 nm in diameter [6]. To explain the rounding, it is probably more appropriate to consider the diffusion of surface atoms rather than complete melting. Another important factor for heat-induced morphological changes, overlooked by many other studies, is annealing time. We believe that the surface effects resulting in the rounding of the NPs are due to energy minimization via a rearrangement of surface atoms, similar to the effect demonstrated recently by Vigonski et al. [17] for heat-induced segmentation of Au nanowires at temperatures much below the melting point of bulk Au. We observed that the effect of rounding was more pronounced for NPs with a five-fold twinned inner structure compared to single-crystalline particles in the form of truncated triangles. This can be explained by the presence of inner stresses stored inside the five-fold twinned NPs and the tendency of such particles to seek mechanisms of stress relaxation [25]. The rearrangement of surface atoms into more rounded outer geometries can be a way of energy minimization while preserving the five-fold twinned inner structure of the NP, as shown in Figure 3 for a Au NP heated to 800 °C.

**Figure 4:**
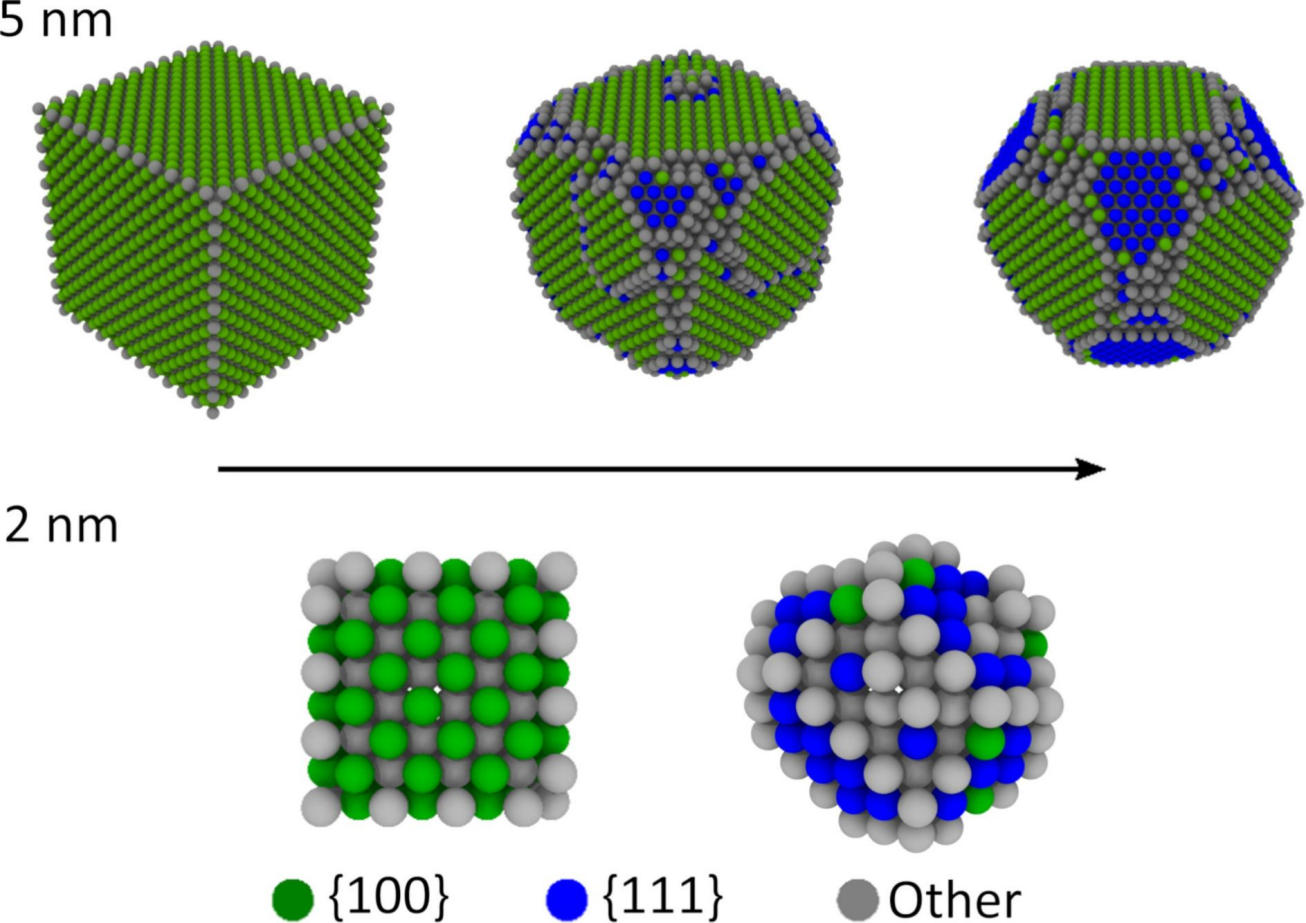
The transition of 5 and 2 nm NPs into rounded shapes bounded by energetically favorable surfaces. Atoms are colored according to their crystallographic surface type.

KMC simulations at 726.85 °C (1000 K) show that the NPs become rounded through a diffusion process which combines the minimization of the surface area and the transition to lower energy surface types (Figure 4). The preferred surface of face-centered cubic (FCC) materials such as Au is {111}, and the simulations clearly show that surfaces of this type grow as the NP relaxes. The KMC model used here describes perfect crystals. Therefore, the resulting shape resembles a dodecahedron bounded by the energetically favorable surfaces {111} and {100}. Our simulations do not include five-fold twinned particles and lower temperatures due to limited computational power and time. Advanced modelling and in-depth theoretical analysis of the rounding process lies outside the scope of the present study and should be addressed separately. Nevertheless, this relatively simple model agrees well with the results of our experiments and sheds light on the processes taking place in small metal particles at elevated temperatures.

### Manipulation of Au nanoparticles

The main goal of this paper was to investigate the effect of the heat-induced rounding of Au NPs on their mobility. Rounding of NPs should decrease the contact area compared to faceted particles, and hence reduce the friction forces in accordance with the known relation τ = *F*/*A* [6], where τ is the contact strength, *F* is the friction force and *A* is the contact area. For a round particle, the contact area is determined by contact mechanics [14,26]. The contact area of a perfect sphere can be two orders of magnitude smaller than that of a polyhedron-like NP, as was shown by Vlassov and co-workers [6].

**Figure 5:**
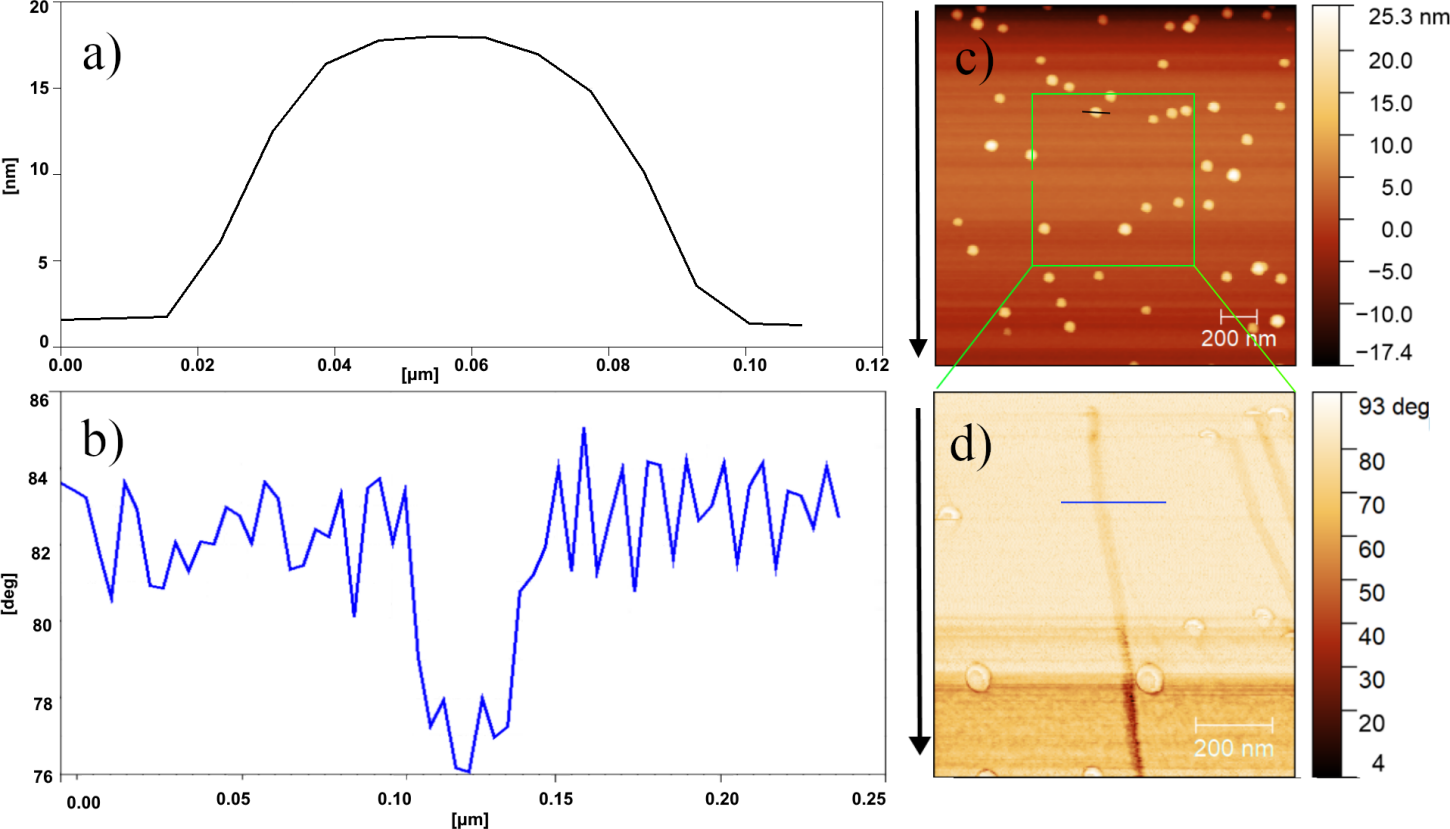
(a) Profile of a Au NP and (b) topography image of NPs on the Si substrate prior to manipulation. (c and d) Phase images recorded during the manipulation.

The mobility of the Au NPs was evaluated by means of the power dissipated in tapping-mode AFM, which has previously been shown to be an effective technique for the evaluation of NP mobility [10]. An example of the manipulation sequences for the sample annealed at 400 °C is given in Figure 5. A selected area is scanned in tapping mode, and the driving amplitude is increased until the displacement of NPs is observed. The dissipated power is then calculated.

**Figure 6:**
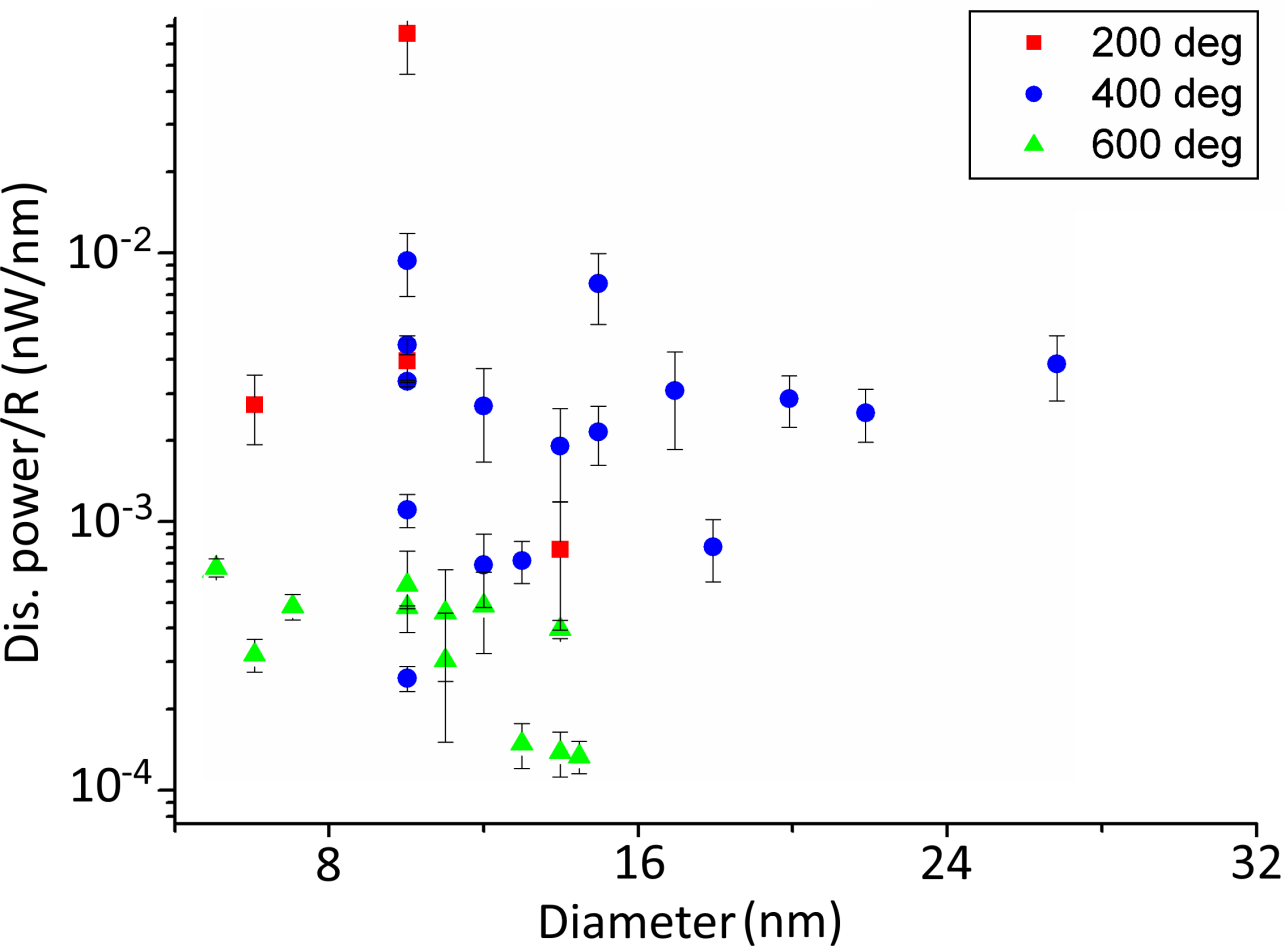
Dissipated power per NP radius as a function of the NP diameter. The diameter is defined as the height of the NP in the AFM image.

In total, 34 data points were collected: 12 measurements for NPs annealed at 600 °C, 16 for NPs annealed at 400 °C and four for NPs annealed at 200 °C (Figure 6). The low amount of data points for the NPs annealed at 200 °C is due to the fact that most of the NPs required more power for a displacement than the experimental setup could provide. Therefore, the actual average friction determined for the NPs annealed at 200 °C is even higher. The power required to displace NPs is the highest for particles annealed at 200 °C and the lowest for NPs annealed at 600 °C. This finding agrees well with the rounding observed by TEM. Moreover, the amount of dissipated power divided by the radius of the NP (power per NP radius) decreases with increasing size of the NP annealed at 600 °C series, which may be attributed to a rolling motion as was demonstrated by Darwich and co-workers [8]. They studied the rolling and sliding motion of NPs as a function of NP size and showed that the dissipated power necessary for sliding has little or no dependence on the NP radius, while the dissipated power needed to provoke rolling decreased clearly with increasing NP radius. The indirect indication of a rolling motion in the present study may serve as additional confirmation of the rounded geometry of the NPs annealed at higher temperatures.

**Figure 7:**
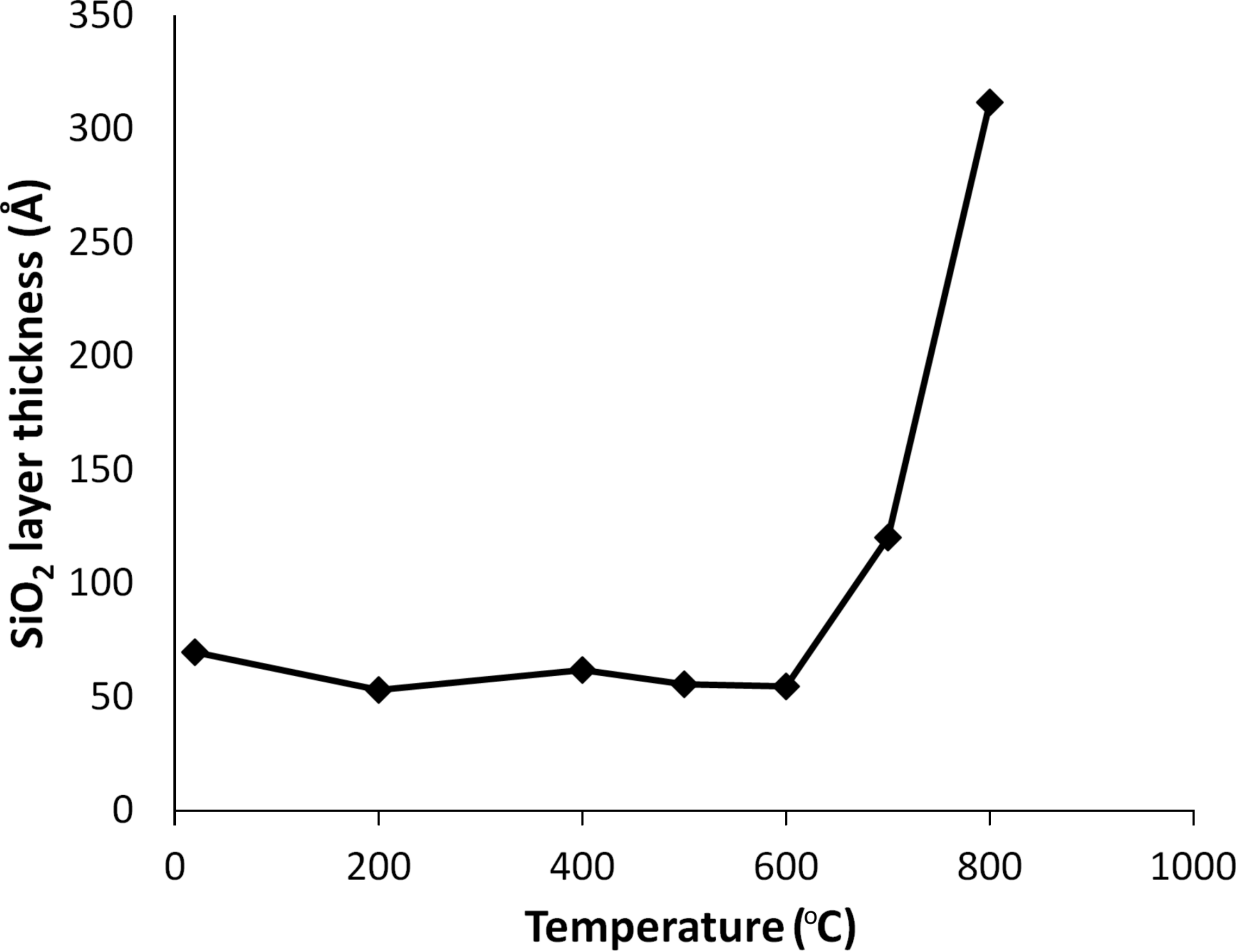
Evolution of the SiO_2_ layer thickness as a function of the annealing temperature.

A displacement of both the unannealed NPs and those annealed at 800 °C was not possible. The immobility of unannealed NPs can be linked to their more pronounced facets (and hence higher surface area) as well as the presence of surfactants that can act as a glue. The immobility of the NPs heated to 800 °C was surprising, especially considering the fact that they look the most round in the TEM images. One possible explanation is related to the partial diffusion of Au into the Si substrate. It is known that gold diffuses into silicon at higher temperatures by the so-called kick-out mechanism, in which self-interstitials present at thermal equilibrium displace substitutionally dissolved Au atoms into interstices, such that these may undergo rapid interstitial diffusion [27]. The process is highly temperature-dependent, and diffusion at 800 °C is four orders of magnitude faster than at 400 °C (see Table 7 in Fisher [28]). Another reason may be the temperature-sensitive growth of the SiO_2_ layer [29], which escalates rapidly above 600 °C, as can be seen from the ellipsometry measurements presented in Figure 7. How exactly the rapid growth of the SiO_2_ layer may be related to the drastic increase in friction remains unclear and can be the subject for future studies. Overall, we demonstrated that heat treatment, which is widely used as a surfactant removal step prior to nanomanipulation experiments, can have extensive effects on the mobility of Au NPs.

## Conclusion

Chemically synthesized Au NPs with a medium diameter of 14 nm were annealed at 200, 400, 600 and 800 °C for 1 h. Untreated particles had irregular faceted shapes. Annealing resulted in geometry changes to a more rounded shape. A slight tendency towards rounding was noticed at 200 °C. The rounding effect became clearly prominent at 400 °C and increased further for higher temperatures. After annealing at 800 °C, most of the particles had sphere-like shapes. KMC simulations at 726.85 °C (1000 K) show that the NPs become rounded by a diffusion process that minimizes the surface energy. The process combines minimizing the surface area and transitioning to the lower energy surface types {111} and {100}. In AFM manipulation experiments, it was found that the higher the annealing temperature, the lower the power necessary to displace the NPs, indicating that the mobility of the particles increases at elevated annealing temperature. However, after treatment at 800 °C, the particles became immovable. We attributed this surprising effect to the diffusion of Au into Si and to the growth of the SiO_2_ layer. Both processes are highly temperature-dependent, exhibiting drastic enhancement above 700 °C. Overall, we demonstrated that heat treatment, which is widely used for surfactant removal prior to nanomanipulation experiments, can have an extensive effect on the mobility of Au NPs.

## References

[1] Liu Y, Sun X, Wang S, Xie M, Chen A, Long R (2012). Mater Lett.

[2] El-Ansary A (2010). Nanotechnol, Sci Appl.

[3] Huang D, Liao F, Molesa S, Redinger D, Subramanian V (2003). J Electrochem Soc.

[4] Thompson D T (2007). Nano Today.

[5] Oras S, Vlassov S, Berholts M, Lõhmus R, Mougin K (2018). Beilstein J Nanotechnol.

[6] Vlassov S, Polyakov B, Dorogin L M, Lõhmus A, Romanov A E, Kink I, Gnecco E, Lõhmus R (2011). Solid State Commun.

[7] Dietzel D, de Wijn A S, Vorholzer M, Schirmeisen A (2018). Nanotechnology.

[8] Darwich S, Mougin K, Rao A, Gnecco E, Jayaraman S, Haidara H (2011). Beilstein J Nanotechnol.

[9] Tong L, Zhu T, Liu Z (2008). Appl Phys Lett.

[10] Mougin K, Gnecco E, Rao A, Cuberes M T, Jayaraman S, McFarland E W, Haidara H, Meyer E (2008). Langmuir.

[11] Rao A, Wille M-L, Gnecco E, Mougin K, Meyer E (2009). Phys Rev B.

[12] Resch R, Lewis D, Meltzer S, Montoya N, Koel B E, Madhukar A, Requicha A A G, Will P (2000). Ultramicroscopy.

[13] Guerra R, Tosatti E, Vanossi A (2016). Nanoscale.

[14] Polyakov B, Vlassov S, Dorogin L M, Butikova J, Antsov M, Oras S, Lõhmus R, Kink I (2014). Beilstein J Nanotechnol.

[15] Grzelczak M, Pérez-Juste J, Mulvaney P, Liz-Marzán L M (2008). Chem Soc Rev.

[16] Polyakov B, Vlassov S, Dorogin L M, Novoselska N, Butikova J, Antsov M, Oras S, Lohmus R, Kink I (2014). Nanoscale Res Lett.

[17] Vigonski S, Jansson V, Vlassov S, Polyakov B, Baibuz E, Oras S, Aabloo A, Djurabekova F, Zadin V (2018). Nanotechnology.

[18] Frens G (1973). Nature (London), Phys Sci.

[19] Grabar K C, Allison K J, Baker B E, Bright R M, Brown K R, Freeman R G, Fox A P, Keating C D, Musick M D, Natan M J (1996). Langmuir.

[20] Anczykowski B, Gotsmann B, Fuchs H, Cleveland J P, Elings V B (1999). Appl Surf Sci.

[21] Jansson V, Baibuz E, Djurabekova F (2016). Nanotechnology.

[22] Baibuz E, Vigonski S, Lahtinen J, Zhao J, Jansson V, Zadin V, Djurabekova F (2018). Comput Mater Sci.

[23] Stukowski A (2010). Modell Simul Mater Sci Eng.

[24] Buffat P, Borel J-P (1976). Phys Rev A.

[25] Dorogin L M, Vlassov S, Kolesnikova A L, Kink I, Lõhmus R, Romanov A E (2010). Phys Status Solidi B.

[26] Derjaguin B V, Muller V M, Toporov Yu P (1975). J Colloid Interface Sci.

[27] Seeger A (1980). Phys Status Solidi A.

[28] Fisher D J (1998). Diffusion in Silicon - 10 Years of Research.

[29] Gorantla S, Muthuvenkatraman S, Venkat R (1998). IEEE Trans Electron Devices.

